# Market-Based Instruments for Biodiversity in Agricultural Landscapes: An Evaluation of Quality Criteria in a German case study

**DOI:** 10.1007/s00267-025-02162-w

**Published:** 2025-04-26

**Authors:** Lea Streit, Arndt Feuerbacher, Markus Röhl

**Affiliations:** 1 Nürtingen-Geislingen University of Applied Science, Institute for Landscape and Environment, Hechinger Str. 12, 72622 Nürtingen, Germany; 2 University of Hohenheim, Ecological-Economic Policy Modelling Research Group, Institute for Agricultural Policy and Markets, Schwerzstr. 46, 70599 Stuttgart, Germany; 3 University of Hohenheim, Center for Biodiversity and Integrative Taxonomy (KomBioTa), Stuttgart, Germany

**Keywords:** Non-governmental schemes, Ecosystem services, Land-use management, Payment for Ecosystem Services, Biodiversity Offset, (Agro-)Ecology

## Abstract

Market-based instruments (MBIs) for the protection and promotion of biodiversity have gained significant importance in recent years. The success of MBIs depends largely on the transparent presentation of their actual effects, which rely on the quality of implementation. Quality criteria can be used to evaluate this. To date, few studies have examined whether and how these criteria are applied. This study seeks to address this gap, by using MBIs in the German agricultural landscape as a case study. Quality criteria were defined on the basis of a literature review; then applied to MBIs identified through an internet search and finally analyzed. Quality criteria related to methodological approaches (implementation, maintenance, used seed mixtures) and quality control (monitoring, localization) are presented less frequently than information on rights and obligations or the use of financial funds. Among the 151 MBIs analyzed, 70% lack control mechanisms and monitoring systems, indicating unverified effectiveness. Additionally, MBIs financed through program approaches are more likely to have control mechanisms and include perennial measures than MBIs funded by direct payments of sponsors or consumers purchasing a product. The development of MBI offerings suggests that there is persistent and growing demand, as some programs have been running for several decades. However, without ecological monitoring, it is not possible to ascertain whether these measures benefit biodiversity. To establish standardized methods for comparing MBIs for biodiversity, policymakers must consider official guidelines and, where appropriate, implement regulatory frameworks.

**Graphical Abstract:**

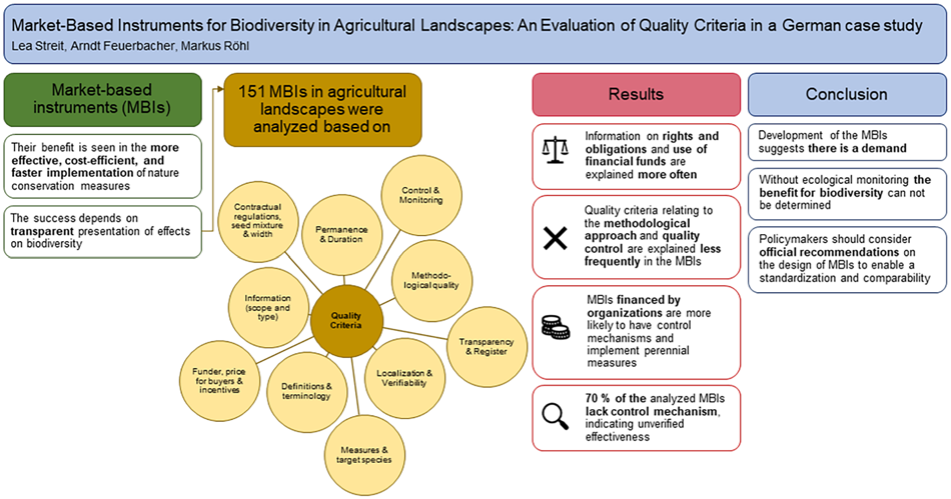

## Introduction

One of the most pressing environmental challenges today is the rapid loss of species across ecosystems (IPBES 2019). Research shows that not only are endangered species declining (Hallmann et al. 2017; Seibold et al. 2019; Burns et al. 2021) but also over 10% of local species richness (global average) has been lost over the last two centuries (Lanz et al. 2018). In addition to climate change, the agricultural sector is considered the most important driver of the decrease in biodiversity (Lanz et al. 2018; IPBES 2019; Cafaro et al. 2022; Whipple and Bowser 2023).

This is partly driven by perverse incentives that favor environmentally destructive practices, such as subsidies for unsustainable agriculture characterized by low crop diversity and high input use, which render biodiversity-friendly farming economically uncompetitive (Pascual and Perrings 2007; Gordon et al. 2015; Convention on Biological Diversity 2023). Biodiversity-enhancing agriculture includes not only organic farming but also conventional farming practices that integrate biodiversity conservation and ecosystem resilience through adaptive management. Local-scale measures, such as establishing flower strips or diversifying crop rotations, can complement landscape-scale interventions like reducing field sizes or incorporating structural elements such as hedgerows (Haines-Young 2009; Tscharntke et al. 2021). Markets continue to undervalue biodiversity and neglect the societal costs of its degradation (Pirard 2012; Gao et al. 2020; Convention on Biological Diversity 2023). Consequently, biodiversity is considered a public good that is universally accessible, leading to insufficient incentives for its sustainable use, as individuals would bear the costs of biodiversity protection while the benefits would be shared globally (eftec and Institute for European Environmental Policy 2010; Convention on Biological Diversity 2023).

There are numerous ways and sources of funding to protect ecosystem services and, particularly, biodiversity. According to Gutman and Davidson (2007), the sources of financing can be divided into (1) public funds (provided by governments), (2) voluntary funds (contributed by conservation NGOs, foundations, or other non-profit organizations), and (3) funds from markets and companies. Among the third source of financing, market-based instruments (MBIs) have gained particular importance in recent years, both in nature conservation and research (eftec and Institute for European Environmental Policy 2010; Pirard 2012; Chobotová 2013; Pirard and Lapeyre 2014). As early as 2007, the European Commission advocated for the increased use of MBIs at local and national levels to achieve environmental and other political goals (Commission of the European Communities 2007). *Decision X/44 Incentive Measures* at COP 10 also encouraged the establishment of MBIs for the protection and promotion of biodiversity (Convention on Biological Diversity 2010).

The term MBI encompasses a wide range of instruments, from cap-and-trade systems, certification schemes, compensation programs, environmental charges, environmental levies, payments for ecosystem services (PES) to subsidies (Chobotová 2013; Pirard and Lapeyre 2014; Gómez-Baggethun and Muradian 2015). A clear definition of MBIs is still lacking, and the term MBI has often been characterized as vague, since many MBIs do not operate within traditional markets (Chobotová 2013; Pirard and Lapeyre 2014; Gómez-Baggethun and Muradian 2015). Pirard (2012) highlights that MBIs share only one common feature: Nature is assigned a price in various forms.

Compared with legally mandated actions, the potential benefit of MBIs lies in their more effective, cost-efficient, and quicker implementation of nature conservation measures (eftec and Institute for European Environmental Policy 2010; Gao et al. 2020). MBIs also foster closer cooperation among different stakeholders (Commission of the European Communities 2007; eftec and Institute for European Environmental Policy 2010; Chobotová 2013; Gao et al. 2020). However, there is criticism that MBIs fail to address the fundamental issue of biodiversity decline, namely a significant change in human and institutional behavior (Gordon et al. 2015).

A significant challenge for MBIs is the frequent absence or inadequacy of independent monitoring provisions, which hinders the quantification of their actual impact (eftec and Institute for European Environmental Policy 2010; Chobotová 2013). However, Chobotová (2013) demonstrated that the transparent presentation of biodiversity impacts is crucial for MBI success. The latter depends on the quality of its implementation, which can be evaluated via quality criteria. In voluntary nature-based climate protection such criteria have been established and are applied by machanisms such as Verified Carbon Standard, Clean Development Mechanism, PeatlandCode, or MoorFutures (Röhl et al. 2022). Similar criteria exist for instruments that address the protection and conservation of biodiversity (cf. Business and Biodiversity Offsets Programme 2012; OECD Environment Directorate 2016; Grimm and Köppel 2019). Although Muradian et al. (2010) noted that a transparent presentation of these criteria in the global MBI implementation process is often lacking.

Since Muradian et al. (2010), additional research has examined MBI characteristics. Sattler et al. (2013) developed a classification system that was applied to 22 German and US case studies, focusing on factors such as actor involvement, voluntariness, and payment terms, with less emphasis on ecological components. Lockie (2013) defined prerequisites for MBIs, such as information clarity and property rights, but did not apply these prerequisites. Grima et al. (2016) applied the classification according to Sattler et al. (2013) to 40 PES cases in Latin America. Grimm and Köppel (2019) proposed quality criteria similar to those used in this study but did not apply them to case studies. Gao et al. (2020) identified criteria for MBI markets, including additionality and monitoring, and concluded that many aspects of MBIs remain poorly understood, presenting opportunities for improvement. Vezzoni et al. (2023) identified 14 criteria for an environmental policy mix framework, with a focus on economic and financial aspects. Bücheler et al. (2024) developed a classification for flower strip sponsorships, providing guidelines for providers and sponsors.

However, no recent studies have addressed the implementation of quality criteria or their operationalization, such as in monitoring, among MBIs. This paper aims to address this gap in the German context. Two research questions drive this study: (a) What MBIs for voluntary, biodiversity-promoting measures in agriculture currently exist in Germany?, and (b) What quality criteria do these MBIs fulfill? To answer these questions, this study applies an exploratory approach based on publicly available secondary data obtained from the internet as explained below.

## Materials and methods

### Identification of suitable MBIs

To determine which instrument qualifies as MBI, the concepts of MBIs were refined, via an international literature search using Web of Science and Google Schoolar. Eight search terms used can be found in Appendix A. Relevant studies were identified and selected via the PRISMA method (Page et al. 2021). A total of 44 studies were identified through database searching, and an additional 25 through other sources. After removing duplicates, a total of 51 studies addressing MBIs related to biodiversity or ecosystem services remained. The literature review revealed MBI types, such as cap-and-trade systems, PES or ecolabeling. These types were used as search terms for a comprehensive internet search. Subsidies and reduced tax rates were excluded, as they are often not cited in the literature as MBIs in the strict sense (cf. Coggan et al. 2009; Hahn et al. 2015). The 20 search terms used (English and German) can be found in Appendix B.

Research on MBIs was conducted via Google between March 21, 2023, and June 12, 2023. The number of search results per search term ranged from a minimum of 60 to a maximum of 190. The search results for five search terms (marked in italics in Appendix B) were not pertinent to the research project as the results were not related to MBIs. In total, 1810 web pages were identified. Each page was reviewed for its relevance to the research questions.

Following an initial screening to confirm whether the page contained pertinent information, 477 potentially relevant pages remained. After removing duplicates, 300 unique pages left for detailed analysis. These pages were further assessed to identify references to MBIs implemented in Germany and to determine whether the instruments promoted biodiversity in agricultural landscapes. Measures not connected to biodiversity, general subsidies, and those designed for urban or private garden contexts, were excluded from the analysis.

**Fig. 1:**
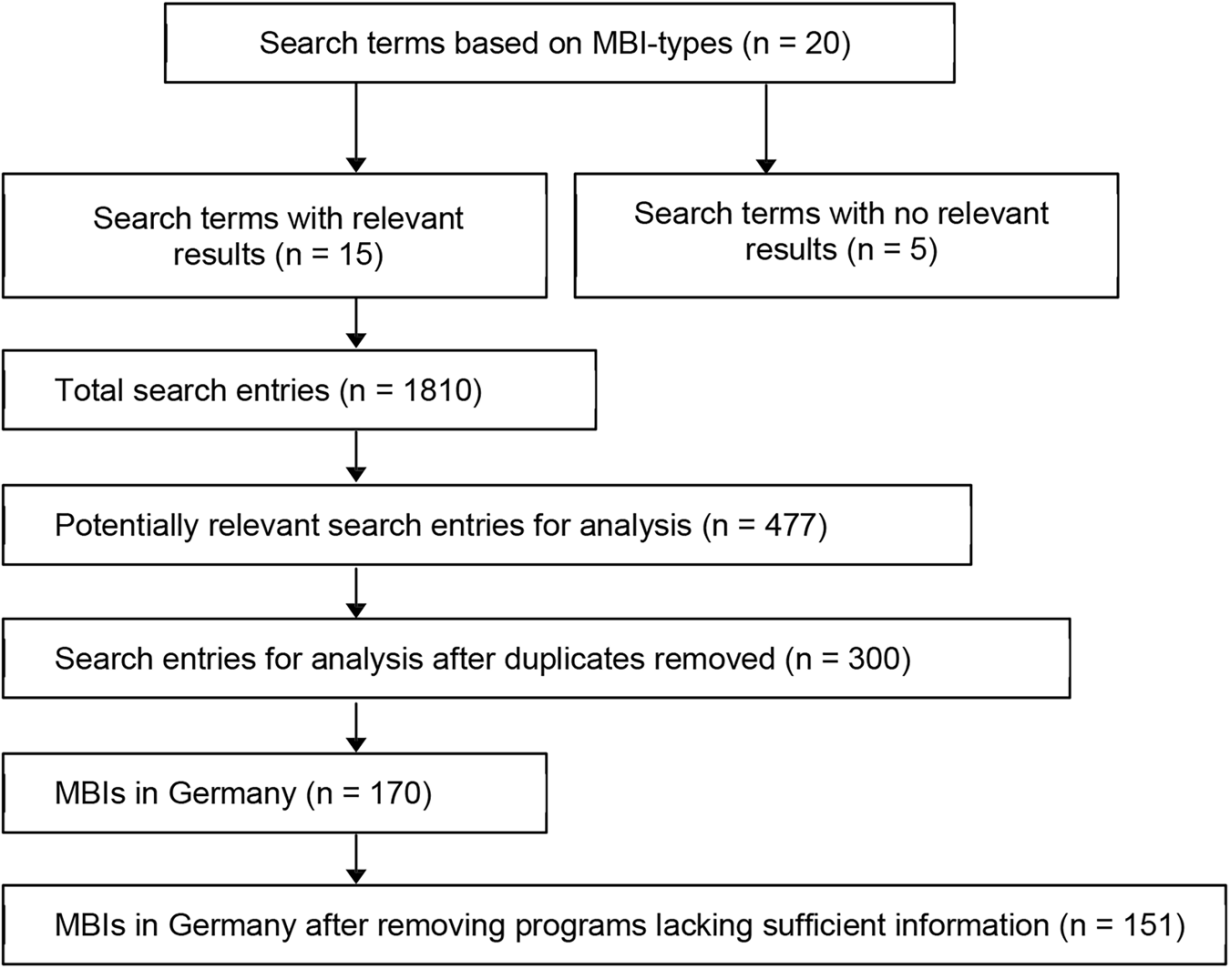
MBI research procedure. The figure illustrates the process for identifying MBIs offered in Germany. Using 20 search terms, a total of 151 MBIs were collected and subsequently analyzed

After completing the screening analysis, 170 MBIs remained for review. Due to being discontinued and lacking sufficient information, 19 MBIs had to be excluded. This resulted in a final sample size of 151 MBIs (Fig. 1).

### Analysis based on quality criteria

The 151 MBIs were analyzed on the basis of established quality criteria. Methodologically, the analysis was guided by the structuring qualitative content analysis approach of Mayring and Fenzl (2019). The fundamental process of this method involves assigning categories to specific text passages based on established rules (Mayring and Fenzl 2019). However, for this study, the method was inverted, and text passages were assigned to a pre-defined category system. This approach allowed for a more structured and systematic analysis of the data, ensuring that all relevant information was captured and categorized appropriately.

The category system was developed by reviewing and synthesizing quality criteria outlined in several biodiversity standards and guidelines, which can be found in the Appendix C. The majority of these guidelines and standards shared common quality criteria but also featured unique aspects. Similar criteria were grouped into clusters, and their frequency across sources was determined. The following clusters were identified, with the number in parentheses indicating the frequency across the reviewed documents: Additionality & No-net loss (9), Permanence & Sustainability (7), Control, Monitoring & Documentation (7), Transparency, Verifiability & Localization (5), Scope (5), Methodological quality (4), Definitions & Terminology (1), Project provider (1). For the analysis, the criteria clusters were refined and supplemented by specific criteria for flower strips and sponsorships, as these comprise 47% of the measures or 61% of the financing types. However, the criteria additionality and no-net loss could not be reliably assessed through internet research; thus, this cluster was excluded from the final analysis. The refined quality criteria used for the analysis are summarized in Table 1.

**Table 1:** Quality criteria and their focus used to analyze the MBIs offered in Germany

Criteria	Description
Permanence & Duration	Period of implementation for the measures.
Control & Monitoring	Availability of information about control or monitoring processes, including details on who conducts these activities.
Methodological quality	Specifications or guidelines for the implementation of measures.
Transparency & Register	Presence of a decommissioning register or an overview of units sold.
Localization & Verifiability	Publication of the location of measure implementation.
Measures & target species	Specific measure or type of biodiversity to be supported.
Definitions & terminology	Use of generally accepted terms.
Funder, price for buyers & incentive for those implementing the measures	Funder of the costs for the implemented measure; price for the funder per hectare or unit; offered incentive to the measure implementer
Information for measure implementers and funders (scope and type)	Publicly available information regarding the implementers’ duties/requirements, compensation, and the use of funds, as well as external communication.
For sponsorship of flower strips: contractual regulations, used seed mixtures & width	Contractual arrangement between sponsor and provider; details of the seeds used; width of the flower strip

The criteria presented form the basis for the qualitative content analysis

The defined quality criteria established the category system. Following the structuring qualitative content analysis approach, the required information was specified for each category (Mayring and Fenzl 2019). Some categories could be standardized with closed answers, whereas others required open responses. The relevant websites or corresponding reports for the 151 MBIs were analyzed. If these sources lacked sufficient information, secondary sources such as newspaper articles or press releases were consulted. Not identifiable information were recorded as ‘not available (n/a)’, not applicable categories were recorded ‘x’.

### Data analysis

The quality criteria established for the content analysis served as the coding scheme for the explorative data analysis. The predefined response options were analyzed using IBM SPSS (version 29.0.1). This approach allowed for a systematic and quantitative evaluation of the data. Possible correlations were explored with the help of cluster analyses and cross tables. Table 2 illustrates the coding process; the link to the complete data set can be found at the end of this article.

The data was analyzed based on the distribution and frequency of the observed values. The sample size for MBIs was *n* = 151, and those related to the measures offered was *n* = 228, as some MBIs provide multiple measures. The distinction between analysis by measure and MBI was based on result significance. For instance, evaluating the permanence of a measure by MBI may not be as informative as evaluating it by measure type, especially when some measures have a greater impact on biodiversity over several years. This approach allowed for a comprehensive understanding of the data, including the identification of any patterns or trends. The analysis of the distribution of the data provided insights into the central tendency of the observed values, while the analysis of the frequency allowed for the identification of any outliers or anomalies.

**Table 2:** Illustrations of the coding framework utilized for the analysis of MBIs

Criteria	Answer option = Code
Permanence	Annual, biennial, perennial, annual to biennial, annual to perennial, n/a
Control / Monitoring	Control, monitoring, control & monitoring, none
Localization	Yes, no, unclear
Measures	Flower Strip, Extensification, Planting, Orchard meadows, Agroforestry, Measure to conserve species, Miscellaneous
Seeds	Native, regional, foreign
Information for funders / implementers	Yes, partly, no, x
Federal State	Bavaria, Baden-Württemberg, Hesse …; Germany-wide, Europe-wide, worldwide

## Results

### What MBIs for voluntary, biodiversity-promoting compensation payments in agriculture currently exist in Germany?

**Fig. 2:**
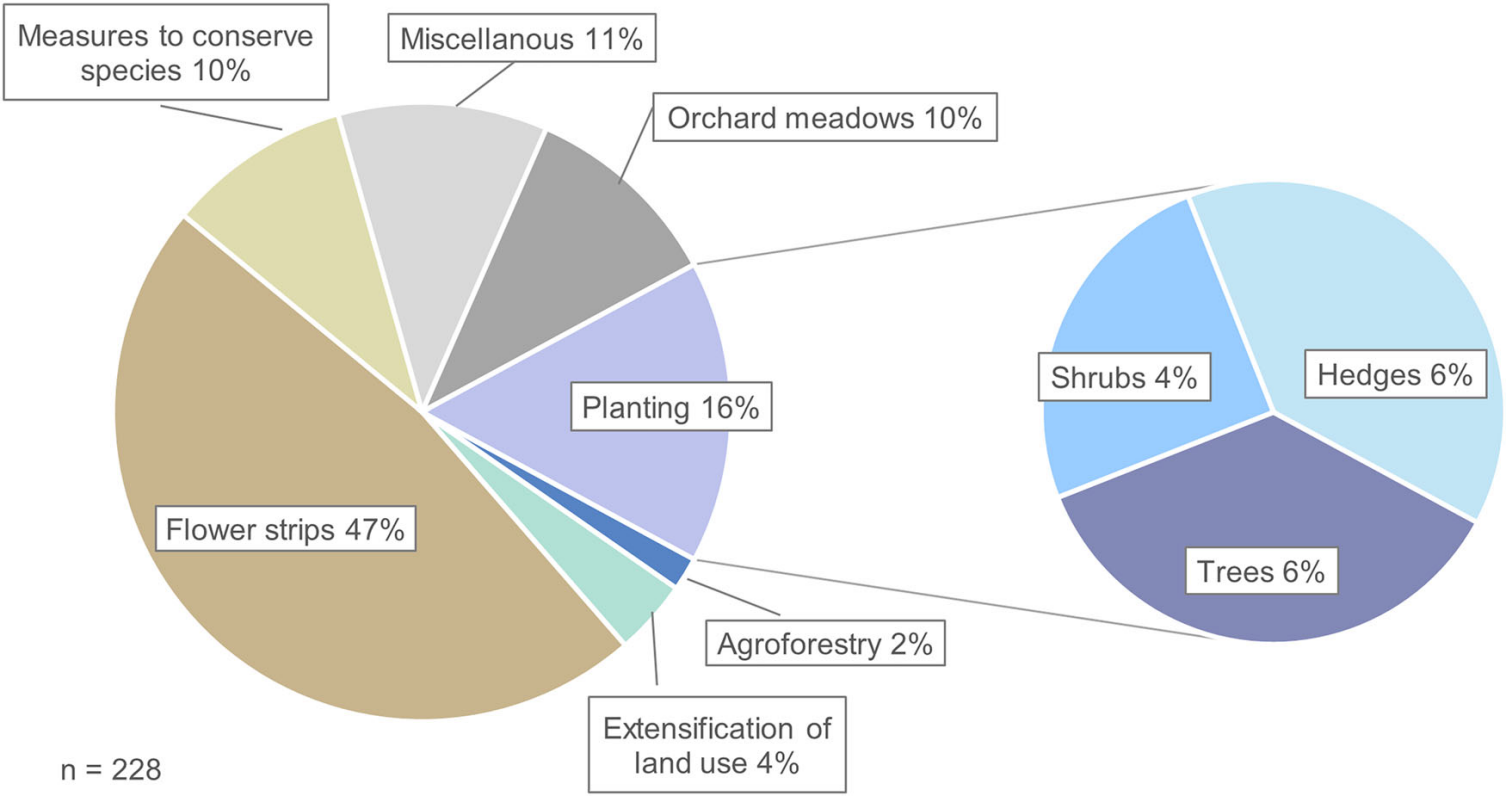
Frequencies of the offered measures (*n* = 228). The planting type is further subdivided into trees, shrubs, and hedges

The 228 measures offered can be categorized into the following types: agroforestry, extensification of land use, flower strips, measures to conserve species, orchard meadows (preservation, maintenance), planting (trees. shrubs, or hedges) and miscellaneous (Table 3). Flower strips were the most frequently offered type (47%), while extensification of land use, agroforestry, and other miscellaneous measures are rarely offered (Fig. 2).

**Table 3:** Description of the measures offered

*Agroforestry* ‘is the purposeful growing or deliberate retention of trees with crops and/or animals in interacting combinations for multiple products or benefits from the same management unit.’ (Nair et al. 2022) Source image: T. Elliot		*Extensification of land use* includes the extensive management of grassland or arable land. Characterized by low levels of fertilization, no use of pesticides, sparse mowing, or limited grazing these practices support rough meadows and pastures, or arable land, to promote endangered field weeds.	
The type *Flower strips* includes all forms in which seed mixtures with a high proportion of herbs are sown on arable land. These can be whole fields or narrow strips, consisting of native wild species, foreign cultivars, or mixtures for biogas.		*Measures to conserve species* include amphibian ponds (see picture), using old livestock breeds, or insect hotels. It also involves *field bird windows*, gaps in crop fields for birds like skylarks to land in dense cereal crops.	
*Orchard meadows*, classified as agroforestry systems, are a historically developed form of extensive fruit growing. Characterized by fast-growing, tall-stemmed, and large-crowned fruit trees in loose stands, each tree remains individually recognizable, often differing in species, variety, and age. Plant protection product usage is rare, and undergrowth is utilized through mowing or grazing (Fischer 2007; Landesanstalt für Umwelt, Messungen und Naturschutz Baden-Württemberg 2024).		*Planting type* comprises all measures involving the planting of woody plants (trees, shrubs, or hedges).	
		*Miscellaneous* measures include humus formation, rewetting of peatlands, deadwood piles (see picture), and others.	

The table provides a brief overview of each type of measure, including an accompanying picture (Source if not stated otherwise: L. Streit)

**Fig. 3:**
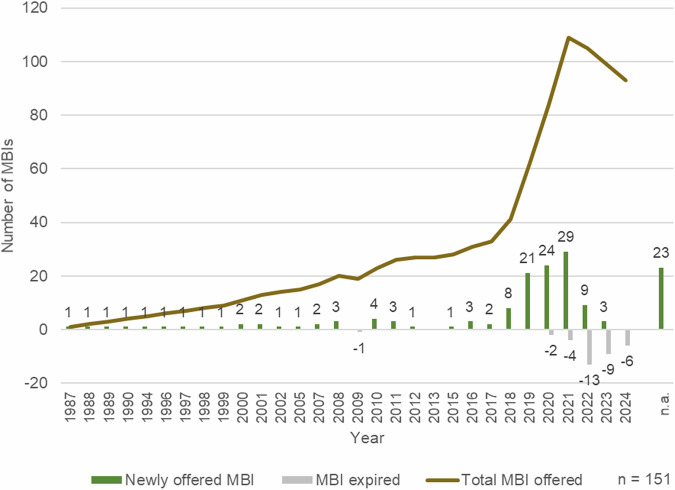
Development of the researched MBI provisions (*n* = 151) since 1987. The brown line represents the progression over time of all MBIs researched. The bars below indicate the absolute number of new or expired programs

In the late 1980s, the first orchard meadow initiatives were launched, particularly in Baden-Württemberg, aiming to preserve these valuable cultural landscape elements (Fig. 3). Until 2017, the number of MBIs offered in Germany increased steadily, with an average of 1.5 programs added each year. From 2018 onward, there was a significant rise in the rate of new MBI offerings, which continued until 2021. On average, 12 new programs were introduced each year during this period, with the sponsorship of flower strips accounting for 74% of the new programs.

Since 2022, the number of programs offered has declined. With more programs being phased out than introduced, the decline averages −3.2 per year. The majority of the terminated offers were sponsorships for flower strips.

**Fig. 4:**
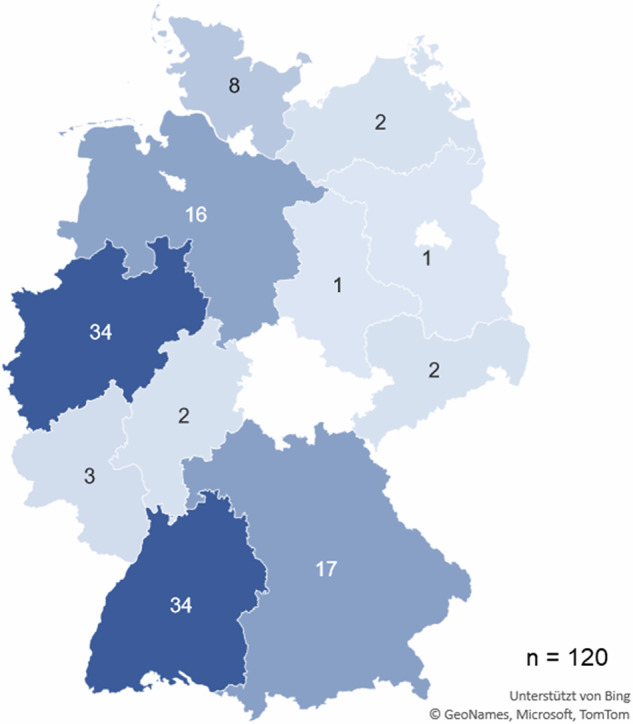
Distribution of local or regional operating MBIs (*n* = 120) across the German federal states

Of the 151 MBIs, 120 or 80% are implemented at the regional or local level and can be specifically assigned to a federal state. Of the remaining 31 MBIs, 26 (17%) operate at the national level and 5 (3%) at the international level (Fig. 4).

### What quality criteria do these MBIs fulfill?

**Fig. 5:**
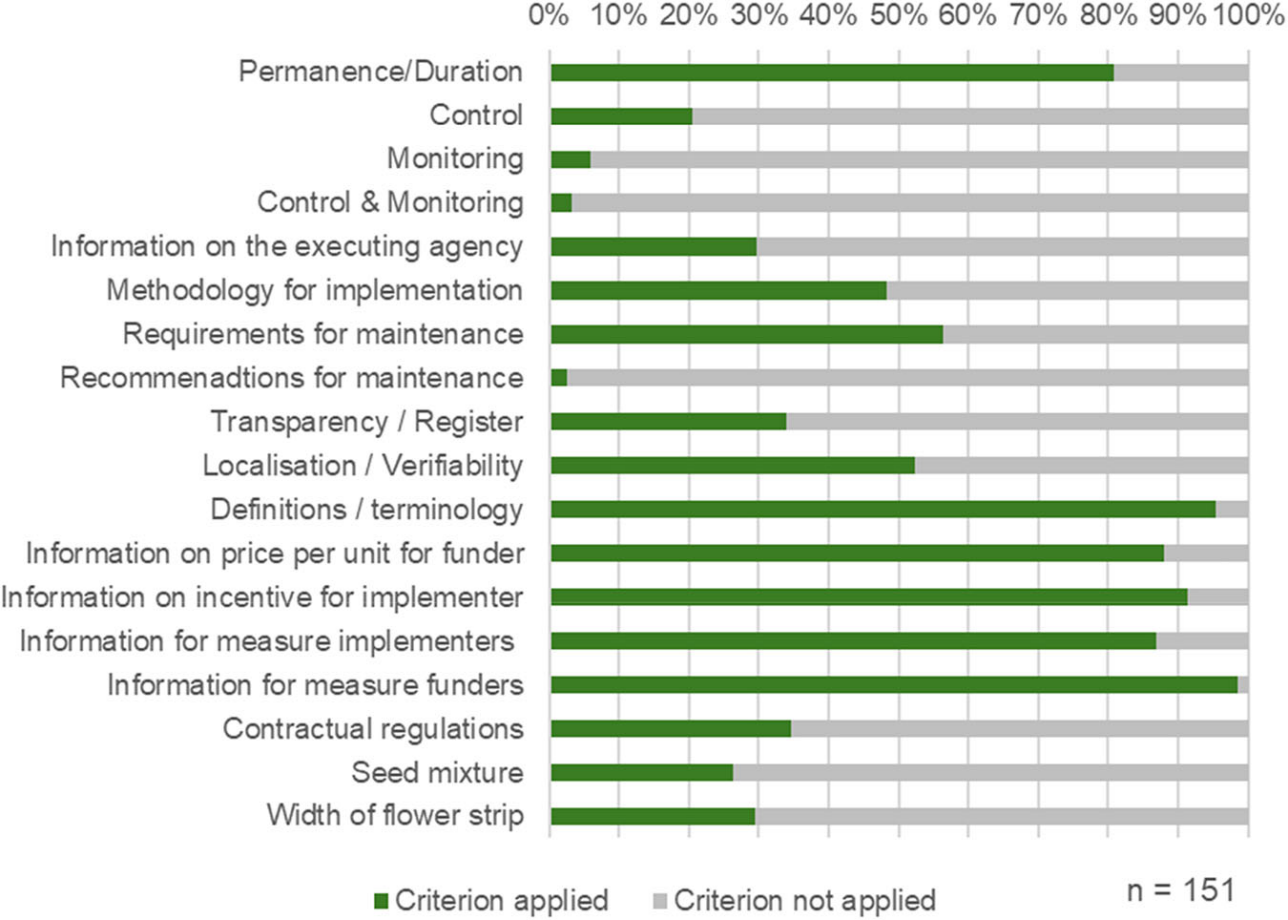
Application of quality criteria in the researched MBIs (*n* = 151)

Quality criteria related to the methodological approach (implementation, maintenance, used seeds, width) and quality control (monitoring, localization, register) are less frequently explained in the MBIs compared to information on rights and obligations and the use of funds (Fig. 5). Information on used seed mixtures or the width of flower strips is only present in less than a third of the MBIs to which this criterion applies.

Measures involving the planting of woody plants, such as agroforestry, orchard meadows, and planting (*n* = 64), are predominantly perennial. In contrast, flower strips (*n* = 108) exhibit a more varied permanence, with 32% being annual or biennial and 27% being perennial. For 20 programs (18%), the permanence is difficult to classify, as both annual and perennial options are offered, while 24 programs (22%) lack information on permanence. Measures to conserve species (*n* = 22) are annual or perennial. The duration of these measures typically depends on their specific nature. For instance, skylark windows are exclusively annual due to the nature of the measure, whereas amphibian ponds are perennial. The measure involving the extensification of land use (*n* = 9) is primarily composed of perennial measures, as is the miscellaneous category (*n* = 25).

The funders were categorized according to the following scheme: sponsors *pay directly* to the measure implementer *in response to an offer*; organizations finance the implementation of measures through *program approaches* in which the measure *implementers participate*; consumers contribute financially by *purchasing a product and creating demand*. The subdivision does not specify what kind of institution provides financial support for the measures. Across all three categories, funding can originate from private individuals, companies or other institutions. However, within the organization category, companies are the dominant providers of programs.

Sponsors finance 61% of the measures, organizations 28% and consumers 11%. A clear pattern can be observed only within the flower strips, which are primarily funded through sponsorships where sponsors pay a one-time amount per square meter to finance seeds and sowing. In return, they receive a sponsorship certificate. For the other types of measures, the funders vary, with organizations more frequently assuming financing. However, no clear patterns can be discerned in the evaluated MBIs.

In MBIs from program approaches, guidelines on the methodological approach and information on the implementation of measures are more frequently provided. Additionally, these measures are more likely to be perennial. Measures financed by sponsors are more frequently located compared to those financed by other funders.

**Fig. 6:**
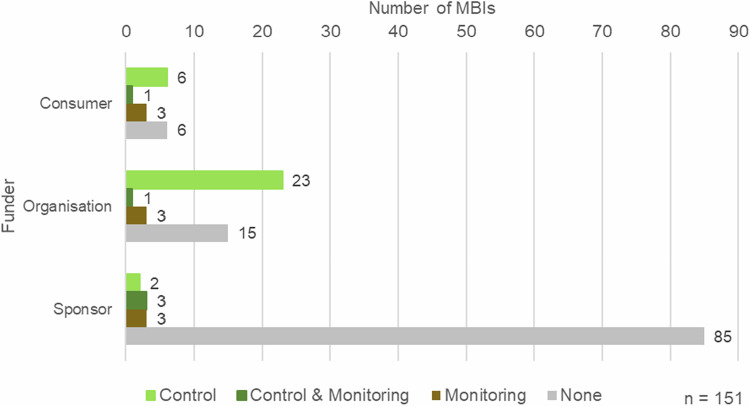
Numbers of MBIs offered with control and/or monitoring mechanisms, categorized by funder (*n* = 151)

In this context, a control mechanism refers to the review of the implementation of measures by an external third party, whereas monitoring refers to ecological supervision. The majority of MBIs, 70% or 106 of the surveyed instruments, have neither a control mechanism nor a monitoring scheme in place (Fig. 6). Three quarters of these 70% are flower strips, with over half (57%) being sponsorships offered directly by farmers.

Among the MBIs analyzed, 36 (24%) have control mechanisms, and five of these also have monitoring mechanisms. Most of these are orchard meadow programs that often require organic certification with regular organic inspections. MBIs with monitoring mechanisms (only monitoring mechanisms = 9, monitoring and control schemes = 5) are often offers that have arisen from subsidized nature conservation projects and are usually scientifically monitored. However, this represents only approx. 9% of the total. No MBI offers several measures but only controls or monitors one or more of them; either there is a control and/or monitoring mechanism for the entire MBI, or there is none at all.

Control or monitoring mechanisms are more commonly found in MBIs from program approaches compared to those financed directly by sponsors. The majority of localized areas are not controlled or monitored, while MBIs with control mechanisms often do not specify the location of their plots.

## Discussion

### MBI situation in Germany

Over the last four decades, especially since 2018, there has been strong growth in MBIs for biodiversity conservation (see Fig. 3). This is correlated with the publication of the so-called Krefeld study (Hallmann et al. 2017), the related “Save the bees” initiatives and the rise in awareness for biodiversity among people. Hallmann et al. (2017) reported a 75% decline in flying insects in protected areas since 1990. In response, multiple federal states launched biodiversity protection initiatives, such as Bavaria (01/2019), Baden-Württemberg (09/2019) or North Rhine-Westphalia (07/2021) (Bund für Umwelt und Naturschutz Deutschland Landesverband Nordrhein-Westfalen e.V. n.d.; proBiene - Freies Institut für ökologische Bienenhaltung (gemeinnützige) GmbH 2019; Bayerisches Staatsministerium für Umwelt und Verbraucherschutz 2024). Despite mixed success, many farmers and individuals offered sponsorships for flower strips. This explains their high proportion and distribution across the federal states (cf. Fig. 4 & Bücheler et al. 2024) and the share of sponsors as funders, as sponsorships are often handled directly between the provider and the buyer. The growth in MBIs may also be attributed to increased biodiversity awareness among individuals. Various studies conducted between 2015 and 2021 across different countries worldwide reported an increase in biodiversity awareness among those surveyed (cf. European Commission 2019; Schleer et al. 2022; Bundesministerium für Umwelt, Naturschutz, nukleare Sicherheit und Verbraucherschutz and Bundesamt für Naturschutz 2023).

The coronavirus pandemic may have enhanced this at the beginning, as people’s awareness for nature and visits to parks and woodlands increased significantly in many countries worldwide during the pandemic (Hynes et al. 2021; Soga et al. 2021). The observed decline in MBI offerings may be attributed to the conflict in Ukraine and the resultant agricultural supply chain disruptions. The significant increase in cereal and vegetable oil prices (Glauben et al. 2022) may have incentivized farmers to shift their production focus back to cereal crops at the expense of flower strips. Simultaneously, the ongoing pandemic has concurrently diminished the willingness to pay for ecosystem services (Hwang 2024). Additionally, rising energy and food costs (Glauben et al. 2022; Berndt et al. 2022 have strained household budgets, making discretionary expenses, such as payments for MBIs more susceptible to cuts.

In general, the development of MBIs in Germany is comparable to that in other OECD countries. Biodiversity-friendly incentives have been increasing steadily since 1980, with taxes, fees, and subsidies accounting for the largest share. PES and Tradable Permits have been increasing steadily since approximately 2000, with biodiversity offsets growing specifically since 2011 (OECD Environment Directorate 2024).

In summary, the growth pattern of MBI points to a consistent demand for these programs, which persists despite the current circumstances. The sustained duration of many programs implies their popularity among supporters, indicating that diverse funding sources are committed to support them.

### Compliance with quality criteria

The results show that quality criteria from the methodological approach are less frequently presented than information on the use of funds. Permanence and control are important criteria influencing a measure’s impact and meaningfulness, as well as effectiveness and success. The control and monitoring of measures serve to control and document success and effectiveness and should therefore also play an important role in an MBI.

Of the MBIs examined 70% lack control or monitoring mechanisms, meaning that most MBIs do not assess their impact on biodiversity. The results indicate that control or monitoring is primarily observed in MBIs where implementers participate through program approaches, thereby falling under the category “funded by organizations.” These group of funders (see chapter “Results” for the definition of funders) tend to support perennial measures and ensure that methodological requirements are met and scientifically monitored. Lindenmayer et al. (2023) emphasize that for MBIs to be effective, they must have solid program designs that include valuable monitoring and timely reporting of essential metrics. This aligns with the growing importance of corporate social responsibility for companies (see Deutscher Bundestag 2017; Donner et al. 2024), where positive contributions to biodiversity can be reported. Independent monitoring helps to avoid conflicts of interest and potential misuse of the program and thus counteracts greenwashing accusations (Lindenmayer et al. 2023).

The results reveal that MBIs funded by sponsors are often localized but rarely monitored, while those with control or monitoring mechanisms are seldom localized. Chobotová (2013) highlighted the lack of public information as a key factor contributing to MBI failures. Additionally, Bücheler et al. (2024) emphasized the importance of communication, such as providing regular updates, to ensure the success of sponsorships for flower strips. To enhance transparency and engagement, some providers invite sponsors to visit the locations of the measures regularly. While such visits may not directly verify ecological value, they allow sponsors to observe the implementation firsthand. Furthermore, while localized measures enable sponsors to visit independently, joint appointments between sponsors and providers can facilitate open communication about both successes and challenges. Research indicates that ecological identity, sense of place, and connection to nature are significant motivators for volunteering (Gooch 2003; Admiral et al. 2017; Ganzevoort and van den Born 2020). Furthermore, a close relationship with nature is linked to a greater willingness to adopt environmentally friendly behaviors and an increased appreciation for non-human species (Gosling and Williams 2010; Samus et al. 2022). Positive experiences during visits to funded measures may enhance one’s identification with the measure and the supported MBI, fostering long-term support that benefits both organizers and the measures themselves (cf. following paragraph). This is evident from findings in Finland, where MBIs that involve stakeholders in the development of their frameworks tend to gain more confidence and support (Hiedanpää and Borgström 2014). However, many MBIs still need to improve in terms of communication and transparency to optimize their offerings and increase their chances of success.

Nearly 50% of the measures are perennial, remaining for at least three years, whereas the rest are annual to biennial, or indeterminate. This is due to the fact that almost 25% of the measures are annual or biennial flower strips. Three main factors likely explain this high proportion. First, farmers may fear flowering mixtures causing weed infestations in neighboring areas (Schütz et al. 2022; Westbrook et al. 2024), leading them to plant annual strips initially to assess their impact. Almost 80% of terminated programs were annual strips or lacked permanence information. Second, annual measures offer flexibility, allowing land to return to arable use the following year. Third, the long-term support of the measures by funders is a critical factor in maintaining the measure. Alvarado-Quesada et al. (2014) have already shown that a major limitation of MBIs is ensuring the long-term financing of maintenance costs. Despite this, the distribution of measures indicates a general willingness to implement long-term and perennial measures. This trend is notable, particularly when the ecological benefit of perennial flower strip plantings is significantly greater if they are only plowed after several years (Tschumi et al. 2015; Buhk et al. 2018; Albrecht et al. 2020).

In addition to flexibility, self-administration could influence the permanence and general implementation of MBIs. Subsidies under the Common Agricultural Policy (CAP) are paid only for defined measures (mostly maintenance) and are often linked to a five-year commitment period. The amounts paid are fixed for the period covered by the respective CAP reform and are adjusted only with the next reform. Additionally, sanctions or repayments can be demanded if requirements are not met (Bundesministerium für Ernährung und Landwirtschaft 2022, 2024). In contrast, MBIs are much more flexible in their design and often involve the creation of new habitats. The need for flexibility in biodiversity offsetting is also emphasized by Bull et al. (2015), as it is essential for increasing the added value for biodiversity, especially as the development of new habitats takes time.

Not only do the implemented measures cover a broad spectrum, but the program structure and the financing of measures also take a wide variety of forms (see “Results”) and are subject to the market principle of supply and demand (cf. Gao et al. 2020). Biodiversity offset programs, now established in numerous countries globally, aim to compensate for impacts caused by a diverse range of projects (OECD Environment Directorate 2024). In addition to those implementing and financing the measures, regulatory authorities are often involved. These authorities establish implementation standards and oversee compliance with them (cf. Victorian Government Department of Sustainability and Environment Melbourne 2011; Landesanstalt für Umwelt, Messungen und Naturschutz Baden-Württemberg 2012; Droste et al. 2022). Examples for biodiversity offset schemes include Ökokonto (Germany), BushBroker (Australia), and Wetland Mitigation Banking (USA). The prices for the units being sold are often established through negotiation between the buyer and seller (Natural Resources Conservation Service n.d.; Victorian Government Department of Sustainability and Environment Melbourne 2009). While these examples are structured instruments with legal requirements involving public stakeholders, other MBIs operate with non-governmental intermediaries or entirely without third-party involvement. When intermediaries are involved as providers, they often specify requirements for seed mixtures or maintenance and manage communication, information exchange, and payment processing. There are numerous examples of sponsorships involving intermediary organizations, such as One Tree Planted (Global), Greening Australia (Australia), or The Gloucestershire’s countryside charity (UK). If there is no intermediary, the implementer provides the measure directly. This direct approach can enhance efficiency and reduce costs by eliminating the need for a middleman. However, it also places more responsibility on the implementer, who must handle all aspects of the measure, including communication, implementation, and accountability. This direct relationship can lead to more personalized interactions, but it may also increase the implementer’s workload and potential challenges in oversight (cf. Bücheler et al. 2024). Sponsorship contracts often include a clause stating that no guarantee is given for the success of the measure. In the absence of funding, the measure can be canceled, and the area can be repurposed for other uses. In both scenarios, the price per square meter is determined by the provider. Grima et al. (2016) showed that this last form of MBI is more successful than MBIs with intermediaries.

In summary, MBIs can vary significantly in their design, with their range of measures extending beyond those of the CAP. Thus, they can serve as a valuable complement to state subsidies, particularly in the context of creating new habitats. But the different funding frameworks can be obstacles, as support for long-term measures is not clarified in advance. However, prices can be adjusted with greater flexibility in response to market conditions than fixed CAP subsidies.

Additionality and no net loss are established as quality criteria for biodiversity offsetting (cf. Business and Biodiversity Offsets Programme 2012; OECD Environment Directorate 2016). However, these two criteria could not be evaluated in this study, as the absence of detailed project descriptions makes it impossible to determine whether the implemented measures result in negative effects elsewhere (cf. Cames et al. 2016). Additionally, it is not possible to assess whether the measures are simultaneously subsidized by the CAP. While subsidies and MBIs on the same area are not inherently mutually exclusive – since MBI payments are not EU funds they do not formally constitute double funding (cf. European Network for Rural Development n.d.; Council of the European Union 2002; Bücheler et al. 2024) – this raises questions regarding additionality. Some providers of flowering sponsorships indicate on their websites that they continue to receive the CAP basic premium for the areas but forgo subsidies via cultural landscape programs. However, such cases are the exception rather than the rule. Regarding the criteria of communication and transparency, there is a clear need for improvement, particularly since the financial support involves funds from private individuals or companies that voluntarily contribute to biodiversity conservation and promotion.

### Limitations of the results presented

Owing to background settings, web logs, and personalized advertising IDs it cannot be guaranteed that the search performed with Google was unaffected by previous search queries. Additionally, Google only lists entries that are listed in its index. The results provide an overview of the current range of voluntary biodiversity-promoting MBIs in Germany but are not exhaustive. The exact number of MBIs in Germany is unclear, and the sample size and representativeness cannot be determined. The large number of different MBI concepts limits comparability. The identified quality criteria were meant to minimize this issue, but information was not always available for all criteria. To obtain an even more comprehensive overview, future research could build on the underlying data and search for specific MBIs in a more targeted manner. A targeted search for specific instruments is conceivable, as is a focus on individual federal states. Nevertheless, the results provide a comprehensive overview of the MBI situation in Germany and contribute to a better understanding of the current standing.

This article has not looked in detail at prices/incentives or measure size. Further studies could focus on whether MBIs represent added financial value for the implementers and to what extent the measures are complementary to government efforts.

## Conclusion

The study investigates the MBIs currently in place in Germany for promoting biodiversity in agricultural landscapes and the quality criteria they fulfill. Based on literature, we established quality criteria for MBIs in agricultural landscapes and researched MBIs schemes for agrobiodiversity conservation. Over the past decade the number of offerings of MBIs has significantly increased. The growth indicates a continued demand for such programs. Overall, 70% of the MBIs lack control and do not assess their ecological effectiveness. The advantage of MBIs lies in their more effective, faster, and cost-efficient implementation of conservation measures. However, without ecological monitoring, it cannot be determined whether these measures really benefit biodiversity. Factors such as habitat connectivity or the landscape context should be considered when assessing the added value of measures for biodiversity. For corporate social responsibility reporting and the growing market for ecosystem services (including biodiversity certificates), the actual measurement of effects is of particular importance. To standardize and compare MBIs in the field of biodiversity, policymakers should consider official recommendations and possibly even regulations on the design of MBIs. Voluntary nature-based climate protection projects, which are predominantly based on clearly defined quality criteria, are a model for this purpose.

## Data Availability

The underlying data set, which was created and analyzed for this study, can be found under: Streit, L., Feuerbacher, A., & Röhl, M. (2025). Market-based Instruments (MBI) to promote biodiversity in agricultural landscapes in Germany (2024-10-30) [Data set]. Zenodo. 10.5281/zenodo.15095008.

## Appendix

**A. Search Terms International Literature Search**

market-based AND instruments AND for AND biodiversity, incentive AND programs AND biodiversity, voluntary AND payment AND biodiversity, markets AND for AND ecosystem AND services, biodiversity AND offsets, compensation AND payment AND agriculture, voluntary AND incentive AND policies AND for AND preventing AND biodiversity AND loss, and environmental AND policy AND tools AND biodiversity

**B. Search terms for MBIs in Germany**

Biodiversity AND agriculture, biodiversity AND seal, biodiversity AND label AND agriculture, biodiversity AND standard AND agriculture, market-based AND instruments AND agriculture, payments AND ecosystem AND services AND agriculture, compensation AND payments AND biodiversity, flowering AND sponsorship, surcharge AND initiative AND agriculture, innovative AND financing AND mechanisms AND biodiversity, financing AND field AND bird AND windows, hedge AND sponsorship, field AND sponsorship, financing AND sparse AND arable, arable AND wild AND herb AND sponsorship, *nature* AND *conservation* AND *compensation* AND *levies, eco-papers* AND *agriculture, wild* AND *arable, conservation* AND *arable, eco* AND *labels* AND *agriculture*.

**Original German search terms**

Biodiversität AND Landwirtschaft, Siegel AND Biodiversität, Biodiversitätslabel AND Landwirtschaft, Biodiversitätsstandard AND Landwirtschaft, marktbasierte AND Instrumente AND Landwirtschaft, Zahlungen AND Ökosystemdienstleistungen AND Landwirtschaft, Ausgleichszahlungen AND Biodiversität, Blühpatenschaft, Aufpreisinitiative AND Landwirtschaft, Innovative AND Finanzierungsmechanismen AND Biodiversität, Lerchenfenster AND finanzieren, Heckenpatenschaft, Ackerpate, Lichtacker AND finanzieren, Ackerwildkrautpatenschaft, *Naturschutzausgleichsabgaben, Ökowertpapiere* AND *Landwirtschaft, Wildacker, Schutzacker, Eco* AND *Label* AND *Landwirtschaft*.

**C. Biodiversity standards and guidelines used to develop a category system**

- Biodiversity Offset Design Handbook & Standard on Biodiversity Offsets (Business and Biodiversity Offsets Programme 2009, 2012)
- Biodiversity Offsets - Effective design and implementation (OECD Environment Directorate 2016)
- Recommendations to improve biodiversity protection in policy and criteria of food standards and sourcing requirements of food companies and retailers (Hammerl et al. 2017)
- Landwirtschaft für Artenvielfalt (Gottwald and Stein-Bachinger 2016)
- Natur^plus^ Standard (Leibniz-Zentrum für Agrarlandschaftsforschung e.V. 2023)
- Freiwillige CO_2_-Kompensationen durch Klimaschutzprojekte (Wolters et al. 2018)
- Blühstreifen ja, aber richtig - Checkliste für die Patenschaft & FAQ: Blühflächen, Blühstreifen, Blühpatenschaften im Ackerland (Landesbund für Vogelschutz in Bayern e.V. 2019, 2023)
